# Anti-Inflammatory mechanisms of the proteinase-activated receptor 2-inhibiting peptide in human synovial cells

**DOI:** 10.1186/1423-0127-18-43

**Published:** 2011-06-17

**Authors:** Ta-Liang Chen, Yung-Feng Lin, Chao-Wen Cheng, Shi-Yun Chen, Ming-Thau Sheu, Ting-Kai Leung, Cheng-Hong Qin, Chien-Ho Chen

**Affiliations:** 1 Department of Anesthesiology, Taipei Medical University Hospital, Taipei, Taiwan; 2 School of Medical Laboratory Science and Biotechnology, Taipei Medical University, Taipei, Taiwan; 3 Graduate Institute of Clinical Medicine, Taipei Medical University, Taipei, Taiwan; 4 Graduate Institute of Pharmaceutical Sciences, Taipei Medical University, Taipei, Taiwan; 5 Department of Diagnostic Radiology, Taipei Medical University Hospital, Taipei, Taiwan

## Abstract

**Background:**

Osteoarthritis (OA) is a degenerative joint disease which affects the entire joint structure, including the synovial membrane. Disease progression was shown to involve inflammatory changes mediated by proteinase-activated receptor (PAR)-2. Previous studies demonstrated that PAR-2 messenger (m)RNA and protein levels increased in OA synovial cells, suggesting that PAR-2 is a potential therapeutic target of the disease.

**Methods:**

We designed a PAR-2-inhibiting peptide (PAR2-IP) by changing an isoleucine residue in the PAR-2-activating peptide (PAR2-AP), SLIGKV, to alanine, generating the SLAGKV peptide. We used it to test PAR-2-mediated inflammatory responses, including the expressions of cyclooxygenase (COX)-2 and matrix metalloproteinase (MMP)-1 and activation of nuclear factor (NF)-κB in human synovial cells. As a control, expressions of COX-2 and MMP-1 were induced by trypsin at both the mRNA and protein levels.

**Results:**

The PAR2-AP increased the expression of COX-2 more dramatically than that of MMP-1. When we treated cells with the designed PAR2-IP, the trypsin-induced COX-2 level was completely inhibited at a moderate concentration of the PAR2-IP. With further examination of trypsin-induced NF-κB activation, we observed sufficient inhibitory effects of the PAR2-IP in synoviosarcoma cells and primary synovial cells from OA patients.

**Conclusions:**

Our study suggests that the PAR2-IP inhibits trypsin-induced NF-κB activation, resulting in a reduction in inflammatory COX-2 expression in synovial cells. Application of PAR2-IP is suggested as a potential therapeutic strategy for OA.

## Background

Osteoarthritis (OA) is a degenerative joint disease in which degradation of the cartilage structure is found. A recent investigation demonstrated the significant involvement of inflammatory processes in OA pathogenesis [1]. Induction of inflammatory factors, such as interleukin (IL)-1β, by hormone disruption and/or other factors was shown to contribute to the disease progression [2,3].

Studies on patients and a mouse model demonstrated a key role of proteinase-activated receptor (PAR)-2 in mediating arthritic inflammation [4-7]. PARs belong to the G-protein coupled receptor family that is activated by serine protease-mediated cleavage of the N-terminus of the receptors [8,9]. Mounting evidence indicated that trypsin cleaves PAR-2 at R^34^↓S^35^LIGKV (in human) to expose a hexameric-tethered peptide that binds to conserved regions in the extracellular second loop of the receptor to initiate signaling [10]. The synthetic peptide (PAR2-AP) corresponding to the tethered ligand domain, SLIGKV, mimics the effects of trypsin in cell lines that naturally express PAR-2. Studies also showed that secreted proinflammatory cytokines up-regulate expression of PAR-2, stimulating more secretion of proinflammatory cytokines and metalloproteinases to enhance inflammatory responses [7,11,12]. When activated, PAR-2 is coupled to nuclear factor (NF)-κB activation in cells [13].

NF-κB is a sequence-specific transcription factor that regulates expressions of numerous genes, including cyclooxygenase (COX)-2 and matrix metalloproteinases (MMPs) [14,15]. NF-κB is constitutively present in cells as a heterodimer, consisting of a p50 DNA-binding subunit and a p65 transactivating subunit. NF-κB is normally found in the cytoplasm in an inactivated state by binding to an inhibitor, such as IκBα. NF-κB activation in response to proinflammatory stimuli involves phosphorylation of IκBα, leading to its proteasomal degradation, which enables NF-κB transcription factors to be translocated to the nucleus [16,17]. Optimal induction of NF-κB target genes also requires phosphorylation of NF-κB proteins, such as p65, in response to distinct stimuli [14].

COX-2 is the key enzyme regulating the production of prostaglandin E2 (PGE2), a central mediator of inflammation. In articular chondrocytes, proinflammatory cytokines such as IL-1β and tumor necrosis factor (TNF)-α synergistically induce COX-2 [18]. Recently, the expression of COX-2 was shown to be induced by the activation of PAR-2 through bacterial infection, or the treatment of either trypsin or PAR2-AP, and mediated inflammation in some cell types [19,20]. Inhibition of COX-2 antagonized trypsin-induced PAR-2-dependent itching in an animal model [21].

MMPs mediate cartilage degradation by specifically cleaving matrix proteins [22]. Studies showed that IL-1β also induces expressions of MMPs [23,24]. There is extensive evidence that among MMPs, MMP-1 (collagenase 1), MMP-3 (stromelysin 1), and MMP-13 (collagenase 3) are particularly involved in the OA process [25,26]. Recent study indicated that activation of PAR-2 with the activating peptide induced a significant up-regulation of MMP-1 in bone osteoblasts [27].

Our previous study showed that PAR-2 is expressed in OA synovial cells without stimulation [12]. Treatment with IL-1β increased PAR-2 expression, which can be repressed by transforming growth factor (TGF)-β through multiple pathways in those cells. To further investigate how PAR-2 can be a potential therapeutic target of osteoarthritis (OA), we designed a PAR-2-inhibiting peptide (PAR2-IP) by replacing an isoleucine residue in the PAR2-AP with alanine, generating the SLAGKV peptide. When synovial cells were treated with the PAR2-IP, trypsin-induced NF-κB activation was inhibited, and the COX-2 level was reduced. Herein, we tested an effective PAR-2-inhibiting peptide, in the hopes of providing a potential therapeutic strategy for OA.

## Methods

### Cell culture

Human synovial cells and chondrocytes were isolated from patients undergoing joint replacement surgery [3,12]. Tissues were cut into pieces (2^~^3 mm^3^). Chondrocytes and synovial cells were released from articular tissues by sequential incubation with 0.1% hyaluronidase (Sigma, St. Louis, Mo, USA) for 15 min, 0.5% proteinase for 30 min, and 0.2% collagenase (Sigma) for 12 h at 37°C in Dulbeccok's modified Eagle's medium (DMEM) (Gibco BRL, Grand Island, NY, USA). After isolation, chondrocytes and synovial cells were individually resuspended in DMEM containing 10% fetal bovine serum (FBS), a 1% penicillin-streptomycin solution, a 1% amphotericin B solution, and 1% L-glutamine, and then incubated at 37°C with 5% CO_2_. The media were changed every 3^~^4 days.

A human synoviosarcoma fibroblast-like synovium cell line, SW982, was cultured in 60-mm diameter dishes in Leibovitz's L-15 medium containing 15% FBS, a 1% penicillin-streptomycin solution, a 1% amphotericin B solution, and 1% L-glutamine at 37°C without CO_2_. The medium was replaced every 1^~^2 days.

### Cell treatments

When cells reached 80% confluence, they were treated with various concentrations of stimulants for a certain time period in serum-free medium for the dose-dependent analysis, or they were treated with a specific concentration of stimulants for various time periods for the time-course analysis. Trypsin was purchased from Gibco. IL-1β was from R&D Systems, Inc. PAR2-AP and PAR2-IP were from Genemed Synthsis, Inc. PAR2-IP was designed by replacing the isoleucine residue in PAR2-AP (SLIGKV) with alanine, generating the SLAGKV peptide.

### RNA extraction and polymerase chain reaction (PCR)

To evaluate the messenger (m)RNA levels of COX-2 and MMP-1, total RNA was extracted from SW982 cells using the Trizol reagent (Invitrogen). Reverse transcription was performed using the oligo dT_18 _primer and MMLV-derived reverse transcriptase as described elsewhere [12].

PCR primers for amplification of specific complementary (c)DNAs were synthesized according to the following oligonucleotide sequences: COX-2 sense, 5'-AAACCTCAGCTCAGGACTGC-3' and antisense, 5'-GGCACTAGCCTCTTTGCATC-3'; MMP-1 sense, 5'-GTCAGGGGAGATCATCGG-3' and antisense, 5'-GCCCAGTACTTATTCCCT-3'; and GAPDH sense, 5'-CAAGGCTGAGAACGGGAAGC-3' and antisense, 5'-AGGGGGCAGAGATGATGACC-3'. The PCR was carried out with 2 μl of template cDNA and 23 μl of PCR buffer containing each primer (0.2 μM), dNTP (2.5 mM), and Taq DNA polymerase (1.25 units) (Takara Bio Inc, Japan). In each PCR, 30 cycles of 30 s at 94°C, 30 s at a primer-specific annealing temperature, and 30 s at 72°C were performed in a Creacon Technology PCR System (Southern Africa). The RNA level of GAPDH was determined in every sample as an internal control. After amplification, the products were visualized by electrophoresis on a 2% agarose gel, stained with ethidium bromide, and illuminated with a UV lamp.

### Cell lysate preparation

Whole-cell lysates were obtained from SW982 and primary synovial cells. Cells were washed with PBS, and then lysed in 50 μl of golden lysis buffer containing 20 mM Tris/HCl (pH 7.9), 137 mM NaCl, 5 mM EDTA, 1 mM EGTA, 10 mM NaF, 1 mM sodium orthovanadate, 1 mM sodium pyrophosphate, 0.1 mM β-glycerophosphate, 2 mM phenylmethylsulfo-nylfluoride (PMSF), 0.8 nM aprotinin,10 nM leupeptin, and 5 mM dithiothreitol. Protein concentrations were determined using a Bio-rad assay.

### Western blotting

Equal amounts of whole-cell lysates were analyzed on 10% sodium dodecylsulfate polyacrylamide gel electrophoresis (SDS-PAGE). After electrophoresis, proteins were transferred to polyvinylidene difluoride (PVDF)-nylon membranes. The membranes were blocked with TBST containing 3% bovine serum albumin (BSA) at room temperature for 1 h, and then incubated with primary antibodies against COX-2 (Millipore) at 1:500, MMP-1 (Chemicon, Inc) at 1:1000, IκBα (Santa Cruz Biotechnology) at 1:1000, phosphorylated (p)-p65 (Cell signaling technology) at 1:1000, and GAPDH (Zymed) at 1:1000 in TBST overnight at 4°C. After being washed with TBST three times, the membranes were incubated with secondary antibodies at 1:10,000 in TBST at room temperature for 1 h. After another three washes, membranes were visualized using an enhanced chemiluminescence detection system (GE Healthcare).

### Statistical analysis

Densities of bands on the gels were quantified by Image J (NIH, USA). Results were normalized to the amount of GAPDH. The mean and standard deviation were used to evaluate COX-2 and MMP-1 expression levels. Student's *t*-test was used for the comparison. The effects of stimulation by trypsin, cytokines, and PAR2-AP on COX-2 and MMP-1 expression levels were analyzed as changes relative to an unstimulated baseline. These analyses were performed individually at least three times. Statistical significance was set to *p *< 0.05.

## Results

### Trypsin induced COX-2 and MMP-1 expressions

**Figure 1:**
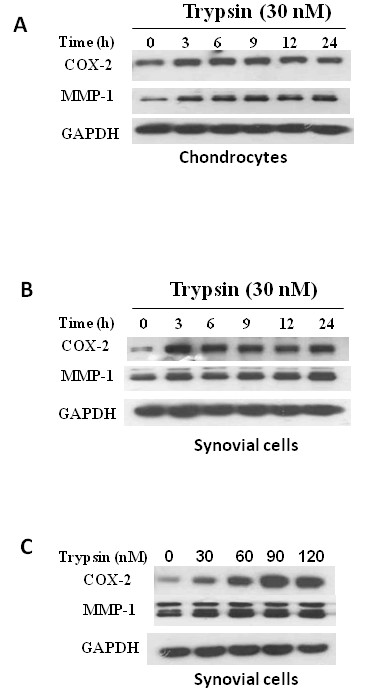
**Induction of cyclooxidase-2 (COX-2) and matrix metalloproteinase-1 (MMP-1) expression by trypsin in human primary cells**. Human primary cells were cultured as described in Materials and Methods. COX-2 and MMP-1 expression levels after trypsin treatment were analyzed by western blotting. Chondrocytes **(A) **and synovial cells **(B) **were treated with 30 nM trypsin in serum-free DMEM for different time periods as indicated. **(C) **Primary synovial cells were treated with various concentrations of trpsin for 8 hours.

Trypsin cleaves PAR-2 and activates inflammatory responses, but it is not clear how COX-2 and MMP-1 expressions are involved in this process in OA patient's cartilage. Therefore, we analyzed trypsin-induced COX-2 and MMP-1 expressions in human primary chondrocytes and synovial cells isolated from patients undergoing joint replacement surgery. Trypsin at 30 nM was able to increase COX-2 and MMP-1 protein levels within 3 h in both cell types; however, the effect was more obvious in synovial cells (Figure 1A, B). This is consistent with higher PAR-2 expression in synovial cells than in chondrocytes reported by a previous study [12]. A further experiment using different concentrations of trypsin demonstrated its dose-dependent effect on COX-2 protein levels in primary synovial cells (Figure 1C).

**Figure 2:**
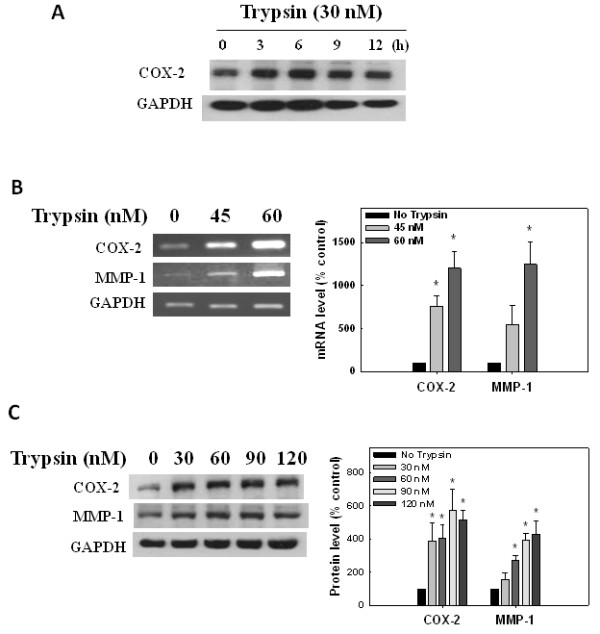
**Induction of cyclooxidase-2 (COX-2) and matrix metalloproteinase-1 (MMP-1) expression by trypsin in human synoviosarcoma cells**. Human synoviosarcoma SW982 cells were treated with trypsin in serum-free L15 medium. COX-2, MMP-1 and GAPDH expressions were assayed by western blotting and RT-PCR. Relative COX-2 and MMP-1 levels were calculated by normalizing the band densities to that of GAPDH and setting the zero controls as 100%. **(A) **Cells were treated with 30 nM trypsin for different time periods. COX-2 and GAPDH proteins were assayed by western blotting. **(B) **After trypsin treatment for 8 hours, COX-2, MMP-1 and GAPDH mRNAs in the cells were analyzed by RT-PCR and agarose gel electrophoresis, and then quantified. **(C) **After trypsin treatment for 8 hours, COX-2, MMP-1 and GAPDH protein levels in the cells were analyzed and quantified.

We then used the human synoviosarcoma SW982 cell line as a model to examine trypsin-induced COX-2 and MMP1 expressions. Similarly we observed an increased COX-2 protein level by 30 nM trypsin within 3 h of incubation in this cell line (Figure 2A). We found that both the mRNA (Figure 2B) and protein (Figure 2C) levels of COX-2 and MMP-1 increased with trypsin treatment, suggesting that trypsin indeed induced the expressions of these two proteins. Dose-dependent effects of trypsin also suggested a close relationship between the trypsin substrate, PAR-2, and the inflammatory genes, *COX-2 *and *MMP-1*.

### PAR2-AP stimulated COX-2 and MMP-1 expressions in synovial cells

**Figure 3:**
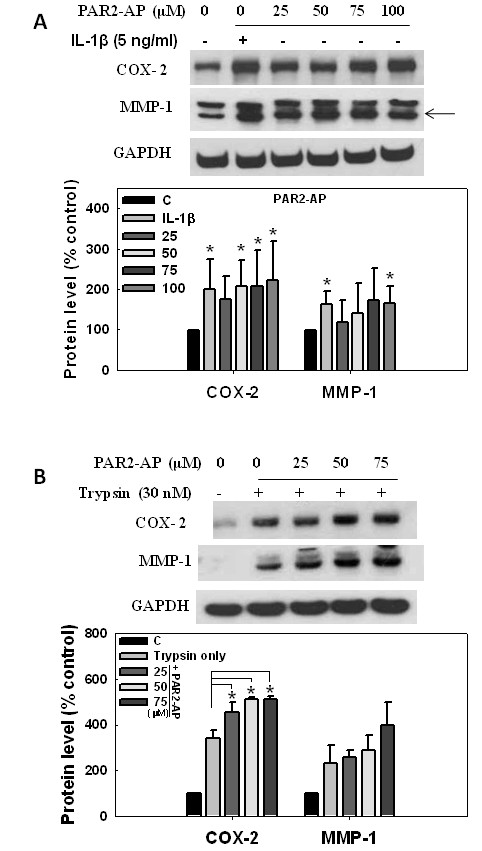
**Induction of cyclooxidase-2 (COX-2) and matrix metalloproteinase-1 (MMP-1)expression by proteinase-activated receptor-2-activating peptide (PAR2-AP) in human synoviosarcoma cells**. Human synoviosarcoma SW982 cells were starved in serum-free L15 medium and treated with the PAR2-AP, IL-1β, and/or trypsin. Expressions of COX-2 and MMP-1 were analyzed by western blotting, and relative levels were calculated by normalizing the band densities to that of GAPDH and setting the zero controls as 100%. **(A) **Cells were treated with IL-1β at 5 ng/ml or PAR-2 AP at different concentrations for 24 hours after 12 hours of starvation. **(B) **Cells were pretreated with PAR2-AP at different concentrations for 30 minutes and then incubated with trypsin at 30 nM for 6 hours.

In chondrocytes, PAR-2 activation by the activating peptide (PAR2-AP), SLIGKV, significantly induced COX-2 and MMP-1 expressions [4]. To test whether the PAR2-AP produces the same effect in synovial cells, we treated SW982 cells with this PAR2-AP at different concentrations for 24 h, and then analyzed COX-2 and MMP-1 protein levels. As a control, IL-1β, which was shown to upregulate PAR-2 expression, increased both COX-2 and MMP-1 levels in cells, suggesting a close correlation between PAR-2 and these two inflammatory proteins (Figure 3A). The PAR2-AP at ≥ 50 μM significantly increased the COX-2 level, but had less effect on MMP-1.

The addition of trypsin to the cells, pretreated with the PAR2-AP, further enhanced the COX-2 level (Figure 3B). These results indicate that PAR-2 activation by PAR2-AP and trypsin leads to COX-2 expression, and PAR2-AP and trypsin had additive effects on this reaction. To our surprise, COX-2 may be more important than MMP-1 in PAR-2-mediated responses in synovial cells.

### The PAR2-IP inhibited trypsin-induced COX-2 expression

**Figure 4:**
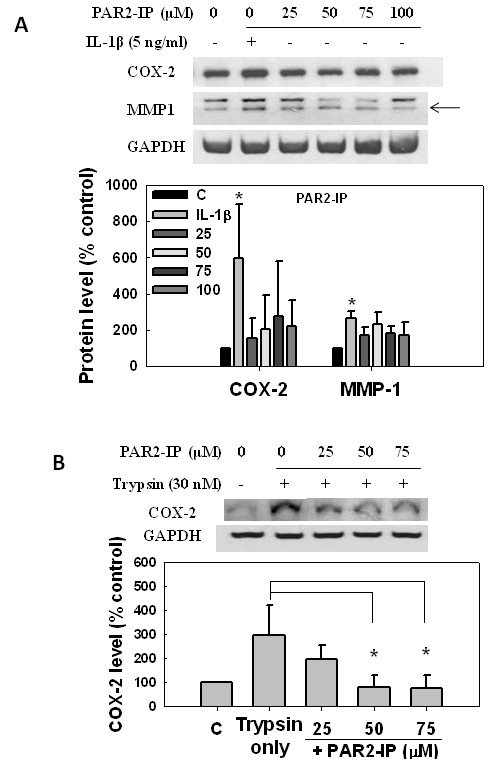
**Inhibition of trypsin-induced cyclooxidase-2 (COX-2) expression by proteinase-activated receptor-2-inhibiting peptide (PAR2-IP) in human synoviosarcoma cells**. Human synoviosarcoma SW982 cells were treated with the PAR2-IP, IL-1β, and/or trypsin in serum-free L15 medium. Expressions of COX-2 and MMP-1 were analyzed by western blotting, and relative levels were calculated by normalizing the band densities to that of GAPDH and setting the zero controls as 100%. **(A) **Cells were treated with IL-1β at 5 ng/ml or PAR-2 AP at different concentrations for 24 hours. **(B) **Cells were pretreated with PAR2-AP at different concentrations for 30 minutes and then incubated with trypsin at 30 nM for 6 hours.

Effects of the PAR2-IP, SLAGKV, on COX-2 and MMP-1 expressions were also evaluated in SW982 synoviosarcoma cells. When treated with the PAR2-IP, cell responses were similar to those with the PAR-AP, but they seemed reduced with PAR2-IP treatment (Figures 3A, 4A).

Since our experiments showed that trypsin induced COX-2 expression (Figures 1, 2), and PAR2-AP pretreatment further increased its level in cells (Figure 3), we examined the effects of the PAR2-IP on changes in trypsin-induced COX-2 expression. It is plausible that the induction was reduced by the additional PAR2-IP in a dose-dependent manner (Figure 4B). The result suggests that the designated PAR2-IP inhibits trypsin-induced COX-2-dependent inflammatory responses in synovial cells.

### The PAR2-IP inhibited trypsin-induced NF-κB activation

**Figure 5:**
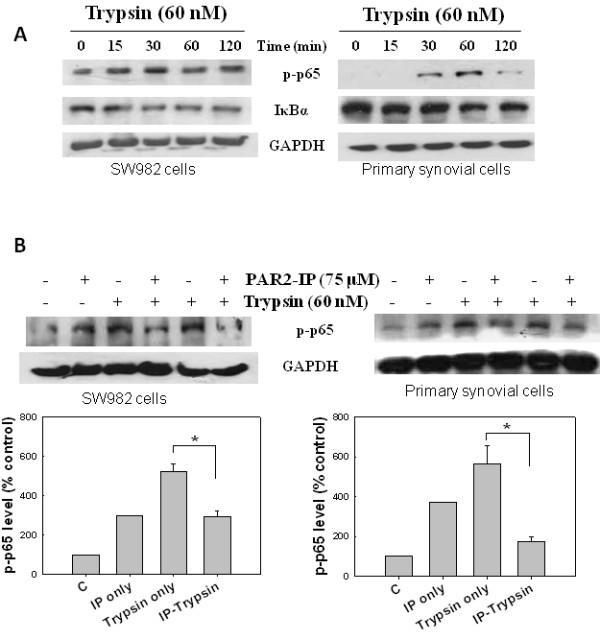
**Inhibition of trypsin-induced nuclear factor (NF)-κB activation by proteinase-activated receptor-2-inhibiting peptide (PAR2-IP) in synovial cells**. Human primary synovial cells or synoviosarcoma SW982 cells were treated with trypsin and/or PAR2-IP, and the levels of phospho-p65 (p-p65), an activated NF-κB, and/or IκBα, an NF-κB inhibitor, were analyzed by western blotting. **(A) **Cells were incubated in serum-free DMEM medium (for primary cells) or L15 medium (for SW982 cells), and then treated with 60 nM trypsin for 15, 30, 60 and 120 minutes. **(B) **Cells were treated with 75 μM PAR2-IP or 60 nM trypsin alone for 30 minutes, or in combination with adding PAR2-IP first for 30 minutes and then trypsin for another 30 minutes. The relative levels of p-p65 were calculated by normalizing the band densities to that of GAPDH and setting the controls as 100%.

It was shown that activated PAR-2 is coupled to NF-κB activation in cells [13], and NF-κB is involved in COX-2 transcriptional activation [14]. We then tested whether the PAR2-IP interferes with NF-κB activation. In control experiments using primary and SW982 synovial cells, treatment with 60 nM trypsin resulted in marked hosphorylation of p65, an activated form of NF-κB, and degradation of IκBα, an inhibitor of NF-κB (Figure 5A). When cells were treated with PAR2-IP alone, phosphorylated p65 levels also increased, a phenomenon that is consistent with the idea that PAR2-IP alone may mimic PAR2-AP on PAR-2 signaling, as seen in Figure 4A. After pretreatment of cells with the PAR2-IP at 75 μM, the trypsin-induced phosphorylation of p65 was inhibited in both cell types (Figure 5B). These results suggest that the PAR2-IP inhibited trypsin-induced activation of NF-κB, which regulates COX-2 expression and inflammatory responses in human synovial cells.

## Discussion

Previous studies showed that PAR2 activation results in proinflammatory effects including vasodilatation, edema, reflux esophagitis, and leukocyte-endothelial interactions [5,28-31]. It was also suggested that luminal proteases activate PAR-2 in the mouse colon to induce inflammation [31]. Following PAR-2 activation, the inflammatory markers, COX-2 and MMP-1, were upregulated in chondrocytes [4]. Our earlier study showed higher expression levels of PAR-2 in human primary synovial cells than in chondrocytes [12]. However, the role of PAR-2 in synovial cells has not been well investigated. Therefore in the present study, we investigated the effects of PAR-2 activation and inhibition of COX-2 and MMP-1 expressions in primary OA synovial cells as well as in model cells, which suggested anti-inflammatory mechanisms of the PAR2-IP.

Trypsin is well recognized as an activator of PAR2. Importantly, trypsin was able to mimic carrageenan/kaolin (C/K)-induced joint swelling, an effect that was abrogated by inhibitors of this proteolytic enzyme [32]. Although there could be a concern of trypsin-induced cell death, similar conditions were used in other studies [13,33]. no sign of increased protein degradation in cells treated with trypsin, and the level of the marker protein, GAPDH, was consistent after trypsin treatment in our experiments. Our study demonstrated that the trypsin-PAR-2 interaction induced COX-2 and MMP-1 expressions in both OA chondrocytes and synovial cells; however, the effect on COX-2 was more obvious than MMP-1 in synovial cells (Figure 1). In primary synovial cells, trypsin induced both COX-2 and MMP-1 protein productions; however, trypsin tended to induce more COX-2 than MMP-1. Likewise this phenomenon was also seen in PAR2-AP-induced COX-2 and MMP-1 expressions (Figure 3). These results suggest that regulation of PAR-2 activity may differ between synovial cells and chondrocytes.

To design the inhibiting peptide, PAR2-IP, we change the isoleucine residue in the PAR2-AP to alanine, generating the SLAGKV peptide. With one residue modification, this peptide has similar effects on PAR-2 signaling; however, it inhibited trypsin-induced COX-2 expression in a dose-dependent manner (Figure 4B). The effect of trypsin was entirely eliminated by PAR2-IP at a moderate concentration (50 μM), suggesting a specific interaction between PAR2-IP and trypsin. Similar phenomena were also seen in trypsin-induced NF-κB activation (Figure 5B). It is known that the sequence of PAR2-AP is identical to trypsin-digested N-terminal PAR-2, and they bind to the same region of PAR2 [10,34]. In other words, PAR2-AP is able to bind trypsin, however, without interference on its activity. Indeed, PAR2-AP and trypsin had additive effects to promote COX-2 expression in the cells (Figure 3B). In the contrary, PAR2-IP may bind to trypsin with high affinity, and consequently inhibits its digesting activity.

Recent studies have demonstrated that trypsin- and PAR2-AP-activated PAR-2 induces inflammatory responses through p65 NF-κB pathway in many cell types. Electrophoretic mobility shift assays, reporter gene assays, and morphological ransduction studies revealed PAR-2-induced activation and translocation of NF-κB in human keratinocytes [13,35]. PAR-2 agonists also activated p65-NF-κB in endothelial and epithelial cells [36,37]. Similarly we found that trypsin activated NF-κB in human synovial cells (Figure 5A). Furthermore our data demonstrated inhibitory effect of PAR2-IP on trypsin-induced activation of NF-κB, and down-regulation of inflammatory COX-2 expression in human synoviosarcoma and primary OA synovial cells.

It was shown that activation of PAR-2 results in proinflammatory reactions via the production of cytokines, such as IL-6, IL-8, and prostaglandin [38,39]. It was also reported that PAR-2 activation induces production of IL-1β and Inter-Cellular Adhesion Molecule (ICAM)-1 by lung epithelial and umbilical vein endothelial cells [40]. Those reports suggested that PAR-2 activation may be associated with local increases in serine proteases that induce cytokine-related inflammation. Although further studies may be required to discover detailed mechanisms, application of PAR2-IP is suggested as a potential therapeutic strategy for OA.

## Conclusions

Our findings suggest that this PAR2-IP inhibits trypsin-induced PAR-2 activation, and represses NF-κB activity, resulting in a reduction in inflammatory COX-2 levels in synovial cells. This is a novel finding that a PAR2-IP can repress NF-κB activation and COX-2 expression. Herein we demonstrated a potential application of a PAR-2 inhibitory strategy that may slow down the OA disease progression and reduce patient symptoms.

## Abbreviations

OA: osteoarthritis; PAR: proteinase-activated receptor; PAR2-AP: PAR-2-activating peptide; PAR2-IP: PAR-2-inhibiting peptide; COX: cyclooxygenase; NF: nuclear factor; IKK: IκB kinase; MMP: matrix matelloproteinase; IL: interleukin; TNF: tumor necrosis factor; TGF: transforming growth factor.

## Disclosure of Potential Conflicts of interests

The authors declare that they have no competing interests.

## Authors' contributions

TLC and CHC conceived of the study and designed research. YFL, CWC and CHC analyzed data. SYC and MTS performed research. TKL and CHQ helped coordinate the study. YFL and CHC wrote the paper. All authors read and approved the final manuscript.

## References

[B1] Pelletier J-P Martel-Pelletier J Abramson SB Osteoarthritis, an inflammatory disease: Potential implication for the selection of new therapeutic targets Arthritis & Rheumatism 2001 44 1237 1247 10.1002/1529-0131(200106)44:6<1237::AID-ART214>3.0.CO;2-F 11407681

[B2] Goldring MB Osteoarthritis and cartilage: the role of cytokines Curr Rheumatol Rep 2000 2 459 465 10.1007/s11926-000-0021-y 11123098

[B3] Wang KC Lin YF Qin CH Chen TL Chen CH Bisphenol-A interferes with estradiol-mediated protection in osteoarthritic chondrocytes Toxicol Lett 2010 198 127 133 10.1016/j.toxlet.2010.06.007 20600708

[B4] Boileau C Amiable N Martel-Pelletier J Fahmi H Duval N Pelletier JP Activation of proteinase-activated receptor 2 in human osteoarthritic cartilage upregulates catabolic and proinflammatory pathways capable of inducing cartilage degradation: a basic science study Arthritis Res Ther 2007 9 R121 10.1186/ar2329 18031579 PMC2246240

[B5] Busso N Frasnelli M Feifel R Cenni B Steinhoff M Hamilton J So A Evaluation of protease-activated receptor 2 in murine models of arthritis Arthritis Rheum 2007 56 101 107 10.1002/art.22312 17195212

[B6] Ferrell WR Lockhart JC Kelso EB Dunning L Plevin R Meek SE Smith AJ Hunter GD McLean JS McGarry F Essential role for proteinase-activated receptor-2 in arthritis J Clin Invest 2003 111 35 41 12511586 10.1172/JCI16913 PMC151840

[B7] Xiang Y Masuko-Hongo K Sekine T Nakamura H Yudoh K Nishioka K Kato T Expression of proteinase-activated receptors (PAR)-2 in articular chondrocytes is modulated by IL-1beta, TNF-alpha and TGF-beta Osteoarthritis Cartilage 2006 14 1163 1173 10.1016/j.joca.2006.04.015 16757188

[B8] Nystedt S Emilsson K Wahlestedt C Sundelin J Molecular cloning of a potential proteinase activated receptor Proc Natl Acad Sci USA 1994 91 9208 9212 10.1073/pnas.91.20.9208 7937743 PMC44781

[B9] Vu TK Hung DT Wheaton VI Coughlin SR Molecular cloning of a functional thrombin receptor reveals a novel proteolytic mechanism of receptor activation Cell 1991 64 1057 1068 10.1016/0092-8674(91)90261-V 1672265

[B10] Dery O Corvera CU Steinhoff M Bunnett NW Proteinase-activated receptors: novel mechanisms of signaling by serine proteases Am J Physiol 1998 274 C1429-1452 10.1152/ajpcell.1998.274.6.C1429 9696685

[B11] L'Hermette MF Tourny-Chollet C Polle G Dujardin FH Articular cartilage, degenerative process, and repair: current progress Int J Sports Med 2006 27 738 744 10.1055/s-2005-872824 16944402

[B12] Tsai SH Sheu MT Liang YC Cheng HT Fang SS Chen CH TGF-beta inhibits IL-1beta-activated PAR-2 expression through multiple pathways in human primary synovial cells J Biomed Sci 2009 16 97 10.1186/1423-0127-16-97 19852794 PMC2773761

[B13] Goon Goh F Sloss CM Cunningham MR Nilsson M Cadalbert L Plevin R G-protein-dependent and -independent pathways regulate proteinase-activated receptor-2 mediated p65 NFkappaB serine 536 phosphorylation in human keratinocytes Cell Signal 2008 20 1267 1274 10.1016/j.cellsig.2008.02.015 18424071

[B14] Viatour P Merville MP Bours V Chariot A Phosphorylation of NF-kappaB and IkappaB proteins: implications in cancer and inflammation Trends Biochem Sci 2005 30 43 52 10.1016/j.tibs.2004.11.009 15653325

[B15] Vincenti MP Brinckerhoff CE Transcriptional regulation of collagenase (MMP-1, MMP-13) genes in arthritis: integration of complex signaling pathways for the recruitment of gene-specific transcription factors Arthritis Res 2002 4 157 164 10.1186/ar401 12010565 PMC128926

[B16] Baeuerle PA Henkel T Function and activation of NF-kappa B in the immune system Annu Rev Immunol 1994 12 141 179 10.1146/annurev.iy.12.040194.001041 8011280

[B17] Grimm S Baeuerle PA The inducible transcription factor NF-kappa B: structure-function relationship of its protein subunits Biochem J 1993 290 Pt 2 297 308 8452515 10.1042/bj2900297 PMC1132272

[B18] Fitzpatrick FA Cyclooxygenase enzymes: regulation and function Curr Pharm Des 2004 10 577 588 10.2174/1381612043453144 14965321

[B19] Lundy FT About I Curtis TM McGahon MK Linden GJ Irwin CR El Karim IA PAR-2 Regulates Dental Pulp Inflammation Associated with Caries Journal of Dental Research 2010 89 684 688 10.1177/0022034510365652 20505052

[B20] Seo JH Kim KH Kim H Role of Proteinase-Activated Receptor-2 on Cyclooxygenase-2 Expression in H. pylori-Infected Gastric Epithelial Cells Annals of the New York Academy of Sciences 2007 1096 29 36 10.1196/annals.1397.067 17405913

[B21] Costa R Marotta DM Manjavachi MN Fernandes ES Lima-Garcia JF Paszcuk AF Quintão NLM Juliano L Brain SD Calixto JB Evidence for the role of neurogenic inflammation components in trypsin-elicited scratching behaviour in mice British Journal of Pharmacology 2008 154 1094 1103 10.1038/bjp.2008.172 18454165 PMC2451046

[B22] Mort JS Billington CJ Articular cartilage and changes in arthritis: matrix degradation Arthritis Res 2001 3 337 341 10.1186/ar325 11714387 PMC128908

[B23] Moos V Fickert S Muller B Weber U Sieper J Immunohistological analysis of cytokine expression in human osteoarthritic and healthy cartilage J Rheumatol 1999 26 870 879 10229409

[B24] Attur MG Dave M Cipolletta C Kang P Goldring MB Patel IR Abramson SB Amin AR Reversal of autocrine and paracrine effects of interleukin 1 (IL-1) in human arthritis by type II IL-1 decoy receptor. Potential for pharmacological intervention J Biol Chem 2000 275 40307 40315 10.1074/jbc.M002721200 11007768

[B25] Tetlow LC Adlam DJ Woolley DE Matrix metalloproteinase and proinflammatory cytokine production by chondrocytes of human osteoarthritic cartilage: associations with degenerative changes Arthritis Rheum 2001 44 585 594 10.1002/1529-0131(200103)44:3<585::AID-ANR107>3.0.CO;2-C 11263773

[B26] Mengshol JA Mix KS Brinckerhoff CE Matrix metalloproteinases as therapeutic targets in arthritic diseases: bull's-eye or missing the mark? Arthritis Rheum 2002 46 13 20 10.1002/1529-0131(200201)46:1<13::AID-ART497>3.0.CO;2-S 11817584

[B27] Amiable N Tat SK Lajeunesse D Duval N Pelletier J-P Martel-Pelletier J Boileau C Proteinase-activated receptor (PAR)-2 activation impacts bone resorptive properties of human osteoarthritic subchondral bone osteoblasts Bone 2009 44 1143 1150 10.1016/j.bone.2009.02.015 19264156 PMC5250314

[B28] Saifeddine M al-Ani B Cheng CH Wang L Hollenberg MD Ratproteinase-activated receptor-2 (PAR-2): cDNA sequence and activity ofreceptor-derived peptides in gastric and vascular tissue Br J Pharmacol 1996 118 521 530 8762073 10.1111/j.1476-5381.1996.tb15433.x PMC1909734

[B29] Vergnolle N Hollenberg MD Sharkey KA Wallace JL Characterization of the inflammatory response to proteinase-activated receptor-2 (PAR2)-activating peptides in the rat paw Br J Pharmacol 1999 127 1083 1090 10.1038/sj.bjp.0702634 10455252 PMC1566112

[B30] Yoshida N Katada K Handa O Takagi T Kokura S Naito Y Mukaida N Soma T Shimada Y Yoshikawa T Okanoue T Interleukin-8 production via protease-activated receptor 2 in human esophageal epithelial cells Int J Mol Med 2007 19 335 340 17203209 10.3892/ijmm.19.2.335

[B31] Vergnolle N Proteinase-activated receptor-2-activating peptides induce leukocyte rolling, adhesion, and extravasation in vivo J Immunol 1999 163 5064 5069 10528212

[B32] Kelso EB Lockhart JC Hembrough T Dunning L Plevin R Hollenberg MD Sommerhoff CP McLean JS Ferrell WR Therapeutic promise of proteinase-activated receptor-2 antagonism in joint inflammation J Pharmacol Exp Ther 2006 316 1017 1024 16260582 10.1124/jpet.105.093807

[B33] Bohm SK Kong W Bromme D Smeekens SP Anderson DC Connolly A Kahn M Nelken NA Coughlin SR Payan DG Bunnett NW Molecular cloning, expression and potential functions of the human proteinase-activated receptor-2 Biochem J 1996 314 1009 1016 8615752 10.1042/bj3141009 PMC1217107

[B34] Holzhausen M Spolidorio L Vergnolle N Role of protease-activated receptor-2 in inflammation, and its possible implications as a putative mediator of periodontitis Memórias do Instituto Oswaldo Cruz 2005 100 177 180 15962119 10.1590/s0074-02762005000900030

[B35] Buddenkotte J Stroh C Engels IH Moormann C Shpacovitch VM Seeliger S Vergnolle N Vestweber D Luger TA Schulze-Osthoff K Steinhoff M Agonists of Proteinase-Activated Receptor-2 Stimulate Upregulation of Intercellular Cell Adhesion Molecule-1 in Primary Human Keratinocytes via Activation of NF-kappa B J Investig Dermatol 2004 124 38 45 10.1111/j.0022-202X.2004.23539.x 15654951

[B36] Syeda F Grosjean J Houliston RA Keogh RJ Carter TD Paleolog E Wheeler-Jones CPD Cyclooxygenase-2 Induction and Prostacyclin Release by Protease-activated Receptors in Endothelial Cells Require Cooperation between Mitogen-activated Protein Kinase and NF-κB Pathways Journal of Biological Chemistry 2006 281 11792 11804 10.1074/jbc.M509292200 16467309

[B37] Wang H Moreau F Hirota CL MacNaughton WK Proteinase-activated receptors induce interleukin-8 expression by intestinal epithelial cells through ERK/RSK90 activation and histone acetylation The FASEB Journal 2010 24 1971 1980 10.1096/fj.09-137646 20065107

[B38] Asokananthan N Graham PT Fink J Knight DA Bakker AJ McWilliam AS Thompson PJ Stewart GA Activation of protease-activated receptor (PAR)-1, PAR-2, and PAR-4 stimulates IL-6, IL-8, and prostaglandin E2 release from human respiratory epithelial cells J Immunol 2002 168 3577 3585 11907122 10.4049/jimmunol.168.7.3577

[B39] Johansson U Lawson C Dabare M Syndercombe-Court D Newland AC Howells GL Macey MG Human peripheral blood monocytes express protease receptor-2 and respond to receptor activation by production of IL-6, IL-8, and IL-1{beta} J Leukoc Biol 2005 78 967 975 10.1189/jlb.0704422 16000389

[B40] Compton SJ Cairns JA Holgate ST Walls AF The role of mast cell tryptase in regulating endothelial cell proliferation, cytokine release, and adhesion molecule expression: tryptase induces expression of mRNA for IL-1 beta and IL-8 and stimulates the selective release of IL-8 from human umbilical vein endothelial cells J Immunol 1998 161 1939 1946 9712064

